# Complications in the treatment with alveolar extraosseous distractors. Literature review

**DOI:** 10.4317/medoral.20512

**Published:** 2015-04-10

**Authors:** Alfredo Rodriguez-Grandjean, David Reininger, Juan López-Quiles

**Affiliations:** 1 DDS. Master in Oral Surgery and Implantology, Complutense University of Madrid; 2 DDS. Master in Oral Surgery and Implantology, Complutense University of Madrid; 3 DDS,MD,PhD. Maxillofacial Surgeon, Associate Professor, Department of Oral Surgery and Maxillofacial Surgery, Complutense University of Madrid

## Abstract

**Background:**

To review the literature that analyses the types and frequency of complications associated with the use of extraosseous alveolar distraction from 2007 to 2013.

**Material and Methods:**

Review of the literature in PubMed, using these keywords; alveolar ridge, alveolar distraction osteogenesis, complication, literature review. Inclusion criteria were: articles published between 2007 and 2013 that included the distraction protocol, the complications encountered and the time when they occurred.

**Results:**

According to the above criteria, 12 articles were included in this review, where 334 extraosseous distractors were placed and 395 complications were encountered, of which 19 (4.81%) were intraoperative, 261 (66.07%) postoperative and 115 (29.11 %) were postdistraction. The most common complication was the incorrect distraction vector found in 105 cases (26.58%), in 23 cases (5.82%) there were severe complications, of which 14 (3.54%) were mandibular fracture and 9 (2.27%) were fractures of the distractor elements.

**Conclusions:**

According to this review, although alveolar distraction is a safe and predictable technique, it can cause complications; however, they are usually minor and easily resolved without affecting the treatment outcome.

**Key words:** Alveolar ridge, alveolar distraction osteogenesis, complication, literature review.

## Introduction

In many situations different anatomical limitations (1) can make implant placement difficult or even impossible. In the case of the upper jaw, some of these limitations are: centripetal resorption of the alveolar process, maxillary sinus pneumatisation, and the presence of the nasal cavity and nasopalatine duct together with bone quality type 3 or 4, in the Lekholm and Zarb classification. For the mandible, the main problem is usually the mandibular canal (2), as well as a decrease in the symphyseal angle which may cause problems in terms of the inclination of the implants. In other situations, despite having enough bone to place the fixture, high bone resorption could lead to an implant placement with an inadequate crown-to-implant ratio so, in addition to compromising the aesthetic outcome, substantial adverse biomechanical forces for these implants would be caused. Currently, there are various techniques to solve these types of defects, such as (3-7): onlay bone grafts, inlay bone grafts, guided bone regeneration and alveolar distraction osteogenesis (DO).

Alveolar DO is defined as the creation of new bone along with adjacent soft tissue after controlled and gradual bone displacement of the bone fragment obtained by surgical osteotomy. This process generates forces that maintain and stimulate regeneration and growth, which is known as the Law of Tension Stress (8,9).

The ability of alveolar DO to regenerate bone and soft tissues makes it an interesting alternative to conventional methods of bone regeneration as it achieves adequate vertical bone augmentation with accompanying soft tissue, optimal aesthetic and functional results, as well as a correct crown-to-implant ratio.

In addition to bone and soft tissue regeneration, the main advantages of alveolar DO are: low infection rate, low resorption, and reduced implant placement time (10). Among the disadvantages, the presence of a high rate of complications are mentioned; however, most of them are simple: difficulty in completing the osteotomy on the lingual side, intraoperative bleeding, paresthesia, hematoma, soft tissue dehiscence, exposure of distractor elements, exposure of the mobilized bone, pain during rod activation, ulcers caused by distractor elements, insufficient attached gingiva (10-26). Severe complications are rare (11-14), with those being: mandibular fracture and fracture of the distractor elements (11,15-20). Several authors mentioned incorrect distraction vector as the most frequent complication (11-14,16-23).

Since the last comprehensive literature review of the complications of alveolar DO by Saulacic *et al*. (15) covered from January 1996 to July 2008, and it did not include all the articles published in 2007 and 2008, the aim of this review is to analyse the types and frequency of complications associated with the use of extra osseous alveolar distraction between 2007 and 2013.

## Material and Methods

The literature review was conducted in PubMed, from January 2007 to December 2013, using the following key words; alveolar ridge, alveolar distraction osteogenesis, complication, literature review.

The selection criteria were: articles which included the number of extra osseous distractors placed per patient, distractor location, distraction protocol, complications, and average total gain. Moreover, articles had to include the time when the complication occurred, which may have been either intraoperative or postoperative, during distraction and/or consolidation periods, and post distraction complications. The following exclusion criteria were considered: studies where intraosseous distractors were used, studies in animals, in patients with any type of bone disease, in patients with a history of facial radiation or treated with intravenous or oral bisphosphonates for more than 3 years.

The side effects caused by the surgery such as pain, haematoma or mucosa inflammation were also included as complications of distraction.

## Results

The initial search resulted in 53 articles, but only 12 of them met the inclusion criteria. In one of these studies (11) both intraosseous and extra osseous distractors were used and complications were not classified according to the type of device. Nevertheless, they were classified according to the time they occurred, so the decision was made to include this article in the review. In these 12 articles, 334 extra osseous distractors were placed, 251 (75.15%) in the mandible and 81 (24.25%) in the maxilla ( Table 1).

**Table 1:**
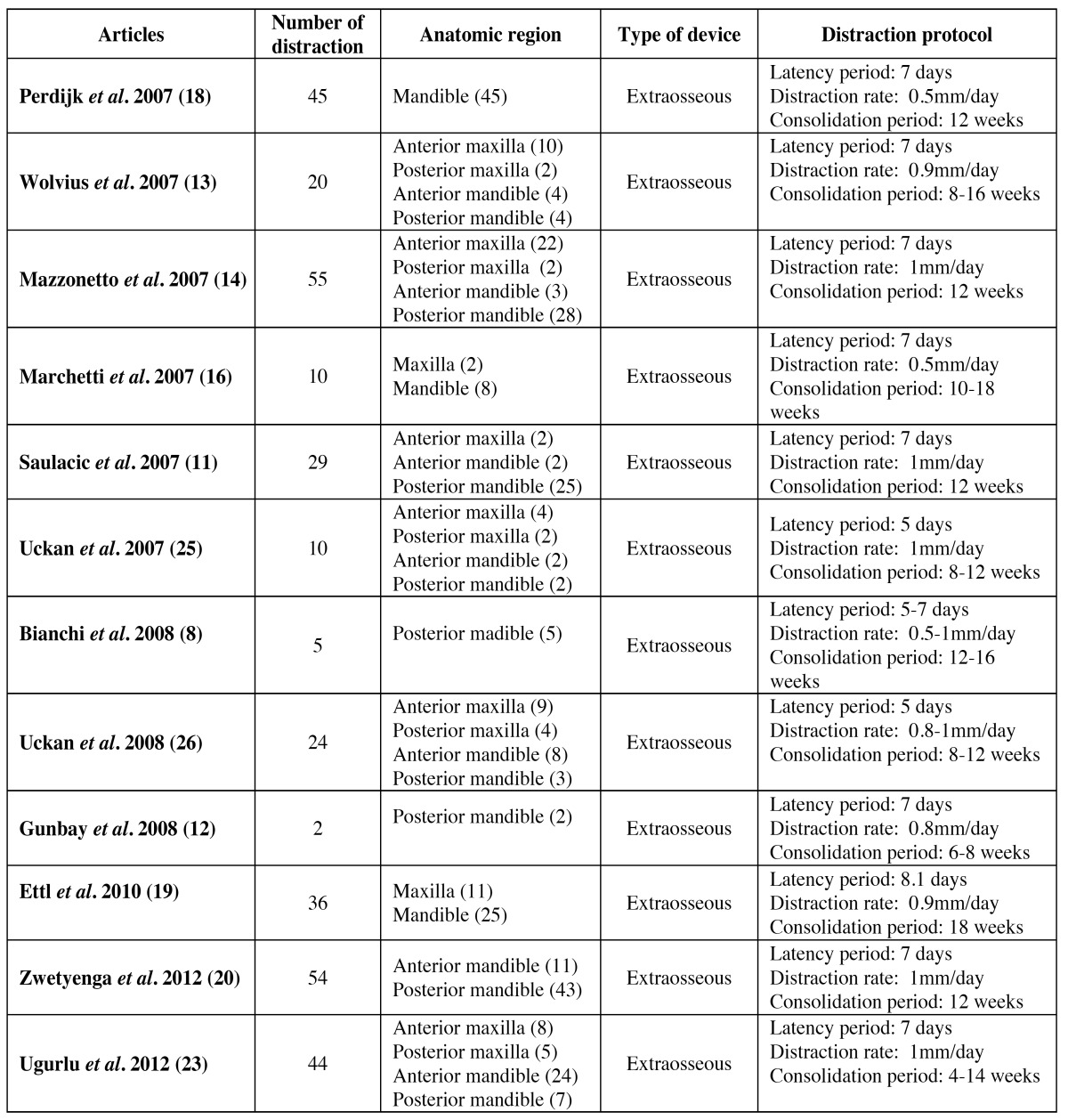
Number of distractors and distraction protocol.

As for the distraction protocol, three parameters were analysed: the latency period, distraction rate and consolidation period. The latency period lasted 5 - 8.1 days, with an average of 6.67 days. The distraction rate was 0.5-1mm/day, 0.85mm/day on average. The consolidation period lasted 4-18 weeks with an average of 11.83 weeks ( Table 1). In total, 652 implants were placed ( Table 2).

**Table 2:**
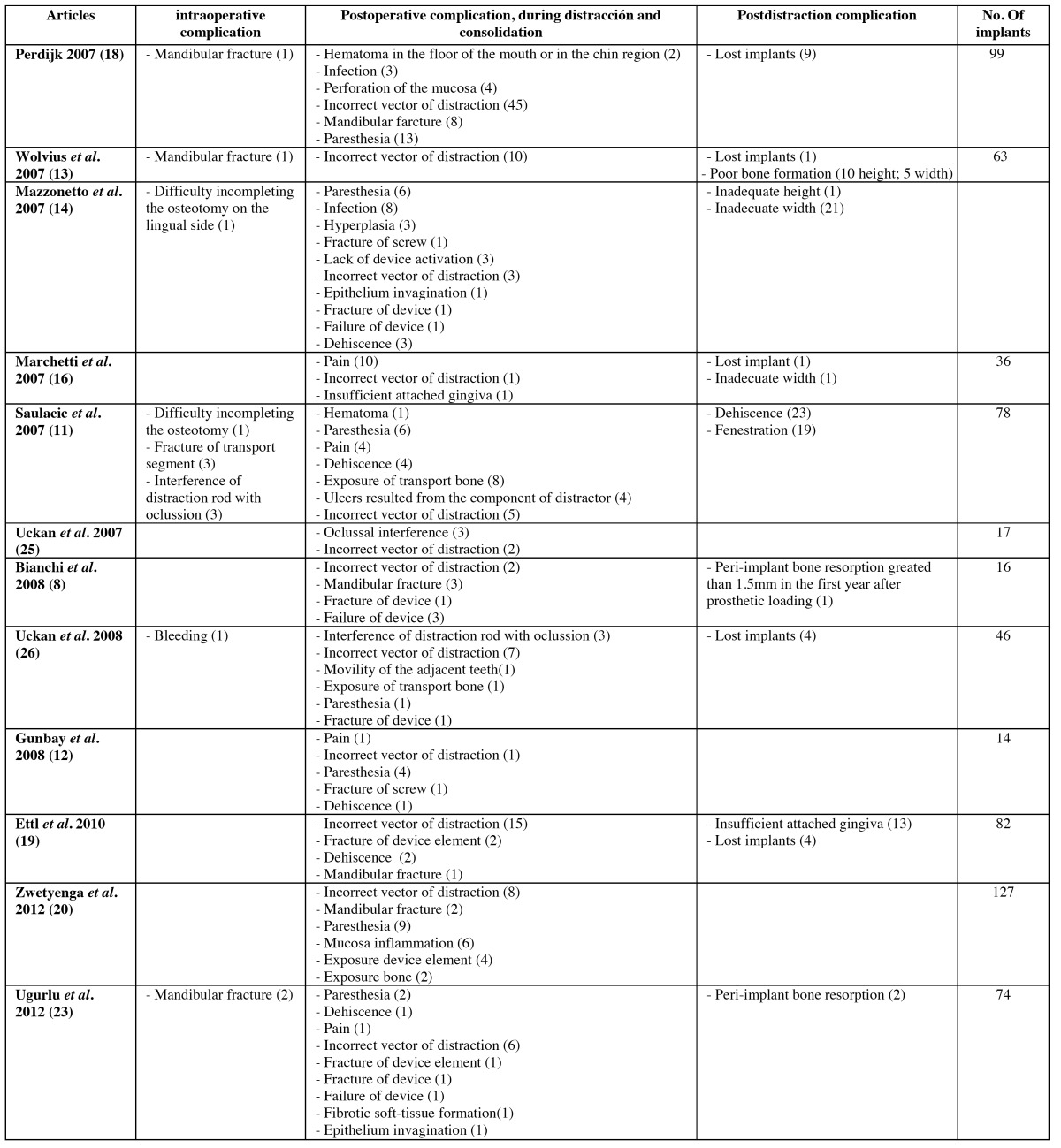
Complications according to time of occurrence.

**Figure 1:**
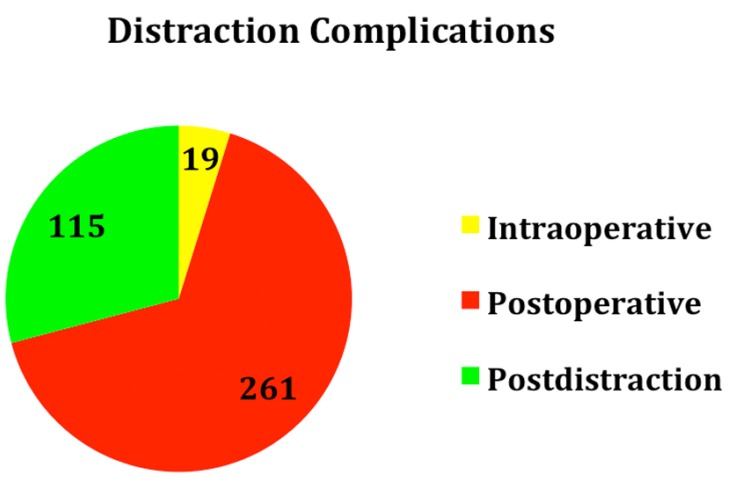
Distraction Complications.

A total number of 395 complications were found, of which 19 (4.81% T, where T is total number of complications in all stages) were intraoperative, 261 (66.07% T) postoperative and 115 (29.11% T) post distraction (Fig. 1).

Of the total cases of intraoperative complications, 8 (42.1%) were due to the difficulty in completing the osteotomy on the lingual side, in 4 cases (21.05%) there was a fracture of the bone to be distracted, in 3 cases (15.78%) a mandibular fracture occurred, in another 3 cases (15.78%) the problem was the rod interfering with the occlusion and finally, in 1 case (5.26%) there was bleeding ( Table 2).

Of the total number of postoperative complications, 105 cases (40.22%) were due to incorrect distraction vector; in 41 cases (15.71%) paresthesia, 19 (7.27%) mucosal dehiscence, 16 (6.13 %) pain, 14 (5.36%) mandibular fracture, 11 (4.21%) infection, 11 (4.21%) due to exposure of distracted bone, 9 (3.44%) fracture of the distractor elements, 6 (2.3%) mucosal inflammation, 6 (2.3%) rod interference with occlusion, 5 (1.9%) distractor failure, 4 (1.53%) ulcers, 3 (1.15%) hematoma, 3 (1.15%) mucosal hyperplasia, 3 (1.15%) inappropriate activation of the distractor, 2 (0.76%) epithelial invagination, 1 (0.38%) adjacent tooth mobility, 1 (0.38%) lack of attached gingiva and 1 (0.38%) because of soft tissue fibrosis related to the rod ( Table 2).

Of all post distraction complications, the most common one was the need for grafting in 27 cases (23.47%) followed by 23 (20%) cases of implant dehiscence. In 19 cases (16.52%) the problem was the failure in implant osseointegration, 19 (16.52%) were due to implant fenestration, 13 (11.3%) lack of attached gingiva, 11 (9.56%) lack of height, so the Tinti technique was used and finally, in 3 cases (2.60%) implants presented peri-implantitis ( Table 2).

The incorrect distraction vector was the most common complication, observed in 105 cases, accounting for 26.58% of all complications, and 40.22% of postoperative complications. Severe complications were found in 24 cases (6.07% T), of which 15 (3.79% T) corresponded to mandibular fracture and 9 (2.27% T) corresponded to fracture of distractor elements.

## Discussion

In recent years, distraction osteogenesis has been established as an effective and predictable method for alveolar bone augmentation, which improves the relationship between the patient’s alveolar ridges. This technique allows a vertical bone augmentation of more than 12 mm, without the need for grafts (22). The main disadvantage is the large number of complications.

Despite being one of the most used techniques in recent years to solve the problem of alveolar defects, certain authors, such as Enislidis *et al*. (17) and Perdijk *et al*. (18), regard DO as a dangerous technique that does not provide advantages over conventional techniques used to increase atrophic alveolar ridges.

If we compare alveolar DO with autogenous grafts to solve vertical bone defects, resorption with autogenous grafts ranges from 25% to 42% (6,27) and the vertical gain is about 5mm (6,8), while with alveolar DO vertical bone gain can exceed 12 mm (8,11,26), with varying degrees of resorption. All authors agree that resorption occurs at the end of the period of consolidation. McAllister *et al*. (28) stated that resorption is not significant, Ettl *et al*. (19) described a resorption of 1.8 mm, Chiapasco *et al*. (22) mentioned a degree of resorption of 0.3 mm, Polo *et al*. (29) 0.9 mm, Jensen *et al*. (30) and Saulacic *et al*. (31) 1. 6 mm.

Despite the many advantages of the alveolar DO already described, such as the ability to regenerate bone and soft tissue, present low infection rate, low resorption and reduce the time in the placement of the implant (10), this technique causes many complications . These range from wound dehiscence to mandibular fracture. In this review, it has been observed that the number of complications increases with the number of cases. The distraction vector is the most common complication, with the following prevalence: 5.45% (14), 10% (16), 13.63% (23), 14.8% (20), 17.2% (11), 29.1% (26), 40% (8), 41.6% (19), 50% (12,13,25) and 100% (18). The tension generated by the lingual / palatal mucosa or by the muscles of the floor of the mouth generates an incorrect inclination of the distraction vector as the fractured fragment is raised (19). This is a very common complication (17,22) that can be solved by the orthodontic replacement (32) of the osteotomized fragment. If the fragment has healed, it is necessary to resort to a new osteotomy and the piece must be placed in the correct position (33,34). To prevent vector displacement, a temporary prosthesis (28) can be used or orthodontic techniques with micro implants (35) during distraction can be performed; alternatively, the bottom of the distractor root can be fixed to the basal bone.

Of all the possible complications that may arise during and after alveolar distraction osteogenesis, mandibular fracture (28,30,36) is the most severe; however, despite being the most severe complication, it does not necessarily imply treatment failure, which surprises us. Many authors suggest that the alveolar DO should be avoided in the cases where mandibular height, measured preoperatively in a panoramic radiograph, is less than 10 mm (18,30) because the risk of fracture is greatly increased. In this review, we have found that Perdjik *et al*. (18) mentioned a total of 9 cases of mandibular fractures, one intraoperative and 8 in the consolidation period, where the residual bone height is less than 10 mm, Zwetyenga *et al*. (20) presented 2 cases, Ettl *et al*. (19) 1 case and Bianchi *et al*. (8) 3 cases, with a total of 15 mandibular fractures (3.79% T). Mandibular fracture was not mentioned in any of the other articles reviewed.

Moreover, fracture of a movable bone fragment was observed during the operation in 4 cases (1.01% T). If the fragment is small, it has to be removed, but if it is big it would have to be fixed with a mini plate. The fracture of a movable bone fragment may generate high residual bone resorption (11).

The fracture of distractor elements or device failure has also been described in this review (8,12,14,19,23,26) and it may determine the success of treatment. In this situation, the procedure should be interrupted and the distractor must be removed as soon as possible.

One case (25,26) (0.25% T) of bleeding in the floor of the mouth during the osteotomy was described; this can be avoided with the use of ultrasound since it reduces the risk of blood vessel injury. However, despite avoiding injury of soft structures and facilitating osteotomy, some authors advocate the use of ultrasound instead of mechanical osteotomy (saws and chisels), which may increase the chance of postoperative and post distraction complications (37).

Paresthesias occurred in 41 cases with a prevalence of 10.38% T, lower than previous data obtained in the literature (14,15), in all cases they were considered minor complications (14) since they were temporary and resolved with conservative treatment.

As for soft tissue complications, dehiscence occurred in 19 cases (4.8%), 11 patients (2.78%) with exposure of movable bone, 6 cases (1.51%) with inflammation of mucosa, 4 patients (1.01%) with ulcers caused by the distractor, 2 patients (0.5%) with epithelial invagination, and 1 case (0.25%) of soft tissue fibrosis. There were also 28 cases (7.09%) with lack of attached gingival, which tend to be more frequent in alveolar DO cases that use extra osseous devices (14,19), since the need to cover the device generates great strain on the mucosa and periosteum (18). These complications do not determine the outcome of treatment and can be resolved with conservative procedures, except for the lack of attached gingival which may require connective grafts (30).

In this review, we noted that the incidence of hematoma and infection were not more relevant than in any other surgery and both are controlled with conservative treatment (11).

Normally, the activation of the distractor rod does not produce discomfort or pain, although in this review, 16 patients (4.05%) reported pain during activation. This can be overcome by reducing the range of distraction from 1 mm to 0.5 mm, which makes discomfort disappear in all cases. However, the average daily range is maintained by increasing the frequency of the adjustments. When performing the osteotomy, it is important to take into account that parallel or converging walls can cause discomfort during distraction and compromise the final result by blocking the mobile fragment, so osteotomies of the side walls should be divergent as recommended by Chiapasco *et al*. (22).

It is possible that the increase in bone increases the risk of dehiscence or fenestration in the vestibular face when placing the implants, and it may occasionally be necessary to use grafts. This is the most frequent post distraction complication, as we have seen in this review, where it was necessary to use grafts in 27 cases (6.83% T). Similarly, we have observed that most of the post distraction complications are related to implant placement. Of the 652 implants, 19 were lost in the early stages of the osseointegration, which gives us a success rate of 97.09%, which is a similar implant success rate compared to natural bone (19,38). In 23 cases (5.82% T) dehiscence occurred, 19 cases (4.81% T) presented fenestration and in 11 cases (2.78% T) there was a lack of height. Multiple studies have demonstrated the occurrence of bone defects at the time of implant placement (11,17,20). One of the causes described was related to the excessive length of the distractor root, as it transmits instability to the mobilized fragment and prevents the formation of new bone at the fracture site. To avoid bone defect problems, callus massages were performed since this technique is included in the distraction protocol because of the good results obtained (39).

Moreover, the narrow alveolar ridge before starting the distraction treatment favours the appearance of fenestrations or dehiscences when implants are placed. However, when the alveolar ridge conditions are unfavorable before starting distraction, bone defects should not be considered as distraction complications, and therefore should not be included in the different studies. Sometimes, when the alveolar ridge is unfavorable, multistage surgery is used by combining bone grafts and distraction (30).

It has been shown that the bone is maintained equally well in implants placed in post distraction bone and those placed in native bone (19).

Mofid *et al*. (40) in a review of 3,278 alveolar DO cases, noted a marked learning curve, with lower rate of complications as the experience of the surgeons increased. Since alveolar DO is a new technique, many complications may be due to lack of experience, inadequate osteotomy, mandibular fracture, inadequate planning, poor device selection and a lack of experience in the surgical management of the distractor placement.

## Conclusions

After analysing the nature of the complications in this review, we consider alveolar distraction osteogenesis as a technique of choice in correcting vertical bone defects because, despite causing multiple complications both during and after surgery, these complications do not usually affect the final outcome of treatment and can be easily resolved. Proper planning, protocols and handling will greatly reduce complications.
